# MicroRNAs: The Link between the Metabolic Syndrome and Oncogenesis

**DOI:** 10.3390/ijms22126337

**Published:** 2021-06-13

**Authors:** Adriana Fodor, Andrada Luciana Lazar, Cristina Buchman, Brandusa Tiperciuc, Olga Hilda Orasan, Angela Cozma

**Affiliations:** 1 Department of Diabetes and Nutrtion, “Iuliu Haţieganu” University of Medicine and Pharmacy, 400012 Cluj-Napoca, Romania; 2 Department of Dermatology, “Iuliu Haţieganu” University of Medicine and Pharmacy, 400012 Cluj-Napoca, Romania; 3 Department of Oncology, “Iuliu Haţieganu” University of Medicine and Pharmacy, 400012 Cluj-Napoca, Romania; 4 Department of Pharmaceutical Chemistry, “Iuliu Haţieganu” University of Medicine and Pharmacy, 400012 Cluj-Napoca, Romania; brandu32@yahoo.com; 5 Internal Medicine Department, 4th Medical Clinic “Iuliu Haţieganu” University of Medicine and Pharmacy, 400012 Cluj-Napoca, Romania; olgaorasan@yahoo.com (O.H.O.); angelacozma@yahoo.com (A.C.)

**Keywords:** metabolic syndrome, adipose tissue, miRNA, cancer, metastases

## Abstract

Metabolic syndrome (MetS) represents a cluster of disorders that increase the risk of a plethora of conditions, in particular type two diabetes, cardiovascular diseases, and certain types of cancers. MetS is a complex entity characterized by a chronic inflammatory state that implies dysregulations of adipokins and proinflammatory cytokins together with hormonal and growth factors imbalances. Of great interest is the implication of microRNA (miRNA, miR), non-coding RNA, in cancer genesis, progression, and metastasis. The adipose tissue serves as an important source of miRs, which represent a novel class of adipokines, that play a crucial role in carcinogenesis. Altered miRs secretion in the adipose tissue, in the context of MetS, might explain their implication in the oncogenesis. The interplay between miRs expressed in adipose tissue, their dysregulation and cancer pathogenesis are still intriguing, taking into consideration the fact that miRNAs show both carcinogenic and tumor suppressor effects. The aim of our review was to discuss the latest publications concerning the implication of miRs dysregulation in MetS and their significance in tumoral signaling pathways. Furthermore, we emphasized the role of miRNAs as potential target therapies and their implication in cancer progression and metastasis.

## 1. Introduction

Metabolic syndrome is a cluster of at least three pathophysiological disorders: abdominal obesity, low-high density lipoprotein (HDL) cholesterol levels, high triglycerides levels, hypertension, and hyperglycemia [1]. The worldwide prevalence varies between 10 and 40%, depending on lifestyle and genetic background [2]. Diet, lifestyle, and genetic background not only affect MetS, but there is increasing evidence showing that these factors play a crucial role in tumorigenesis. MetS has become a predominant risk factor for many cancer types.

Abdominal obesity represents the central feature of MetS and it is associated with alterations in immunity and chronic low-grade inflammation. These can lead to the dysfunction of adipose tissue homeostasis, insulin resistance (IR), macrophages infiltration and polarization, and the release of inflammatory cytokines from both adipose and immune cells. Macrophages are the main immune cells contributing to the activation of inflammatory pathways in obesity and other metabolic conditions. The metabolic and inflammatory changes in adipose tissue can disrupt physiological homeostasis systemically, with initiation and progression of metabolic syndrome and cancer.

Evidence of the last decade supports the crucial role of inflammation in tumor development, progression, and immunosuppression. Most cancers are associated with chronic inflammation induced by environmental factors, such as asbestos, tobacco smoking, and dietary factors, or by chronic bacterial and viral infections, like cervical carcinoma, liver and gastric cancer [3]. Inflammation has been recognized as an essential tumorigenic factor, but it is also often present in the microenvironment of cancers without inflammatory origins [3]. Obesity produces chronic inflammation and an altered profile of key transcription factors that promote a procarcinogen microenvironment. People with obesity have a higher risk of many types of cancers, such as esophagus, gastric, colon, rectum, prostate, liver, prostate, kidney, ovary, meningioma, multiple myeloma, thyroid [4].

Adipose tissue is a complex endocrine organ secreting not only hormones and cytokines, but also a large number of circulating miRs. Adipose tissue derived-miRs from people with obesity have proved essential role not only in obesity-associated inflammation and IR, but in tumor growth and metastasis. Specific miR may act both as miR oncogenic (oncomiR) by suppressing tumor suppressive mRNAs and tumor-suppressive molecule, by suppressing oncogenic mRNAs.

This systematic literature review aimed to revise recent publications on the role of obesity or MetS microRNAs deregulation in adverse prognosis of cancer. Furthermore, we highlight the potential therapeutic role of adipose tissue-derived miRNAs.

We reviewed all publications from the PubMed database using the terms:

(((((“obesity”[MeSH Terms]) OR (“insulin resistance”[MeSH Terms])) OR (“metabolic syndrome”[MeSH Terms])) AND (“neoplasms”[MeSH Major Topic]))) AND (micrornas[MeSH Terms]).

Each relevant microRNA was included in the present analysis if it fulfilled both searches:

1. (“neoplasm invasiveness/etiology”[MeSH Terms]) AND specific microRNA,
2. ((“obesity”[MeSH Terms]) OR (“insulin resistance”[MeSH Terms])) OR (“metabolic syndrome”[MeSH Terms]) AND specific microRNA.

## 2. MicroRNAs Link the Metabolic Syndrome and Cancer

**Figure 1:**
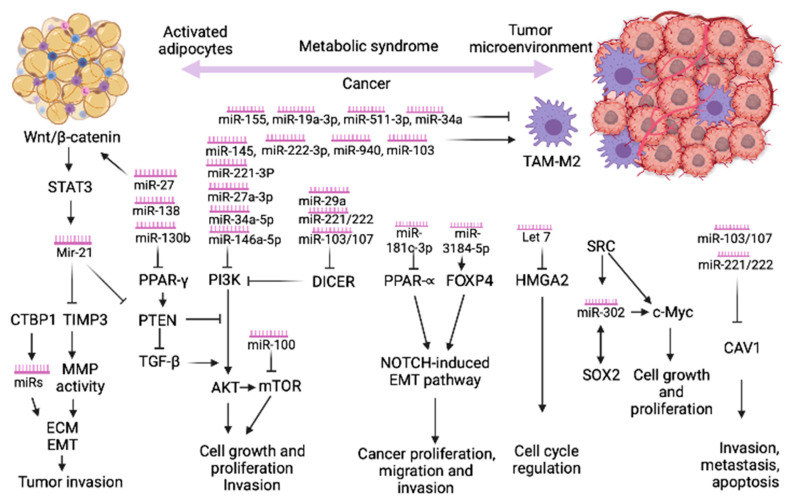
Signaling pathways and the corresponding miRs implicated in MetS- associated cancer. Several miRs are deregulated in both MetS/obesity and various types of cancers. Mir-21 is regulated by B-catenin via STAT3 and plays a role in oncogenesis and cancer progression through its direct targets TIMP3, which modulates matrix MMP activity and the PTEN/PI3K/AKT signaling pathway. PI3K/AKT represents the prevalent pathway implicated in carcinogenesis and MetS/obesity. The Mir221/222 cluster is implicated in both IR and cancer via CAV1, a key regulator for cell proliferation, apoptosis, migration, and metastasis. Other pathways implicated in cancer initiation and progression are: NOTCH-induced EMT, HMGA2, SOX2, c-Myc. TAM—tumor associated macrophages, MMP—matrix metalloproteinases, EMT—epithelial–mesenchymal transition, ECM–extracellular matrix, STAT3—signal transducer and activator of transcription 3, TIMP3—tissue inhibitor of metalloproteinase 3, PPAR-γ—peroxisome proliferator-activated receptor gamma, PTEN–phosphatase and tensin homolog, PI3K—phosphoinositide 3-kinase, AKT—protein kinase B, mTor—mammalian target of rapamycin, PPAR-α—Peroxisome proliferator activated receptor alpha, HMGA2—high mobility group AT-hook 2, SRC—Proto-oncogene tyrosine-protein kinase Src, SOX2—SRY-Box transcription factor 2, c-Myc—Cellular myelocytomatosis oncogene, CAV1—Caveolin 1.

MicroRNAs (miRs) are small (about 18–25 nucleotides), non-coding RNAs, which negatively regulate gene expression by translational inhibition or mRNA decay. miR is associated with Argonaut proteins and incorporated into the miR-induced silencing complex (mRISC), which guides the binding of miRs to the 3′UTR of the target mRNAs. Due to short binding sequence, with imperfect complementary, an individual miR can bind and affect the expression of hundreds of mRNAs [5]. In addition, miRs have been found to be secreted to the extracellular space as membrane-covered microvesicles, such as exosomes, which can be taken up by neighboring or distant recipient cells. Adipocyte-derived microvesicles contain about 140 miRs, while in adipose tissue, macrophages-derived exosomes are identified in about 500 miRs [6,7]. Several pathways, controlled by miRs, have been proposed in the last decade to explain the increased risk of cancer in the context of MetS. We emphasize below the most relevant ones (see also Figure 1).

### 2.1. The Role of miRNAs in Cancer by Modulating Macrophage Phenotypes

The inflammatory tumor microenvironment contains both innate immune cells (like, macrophages) and adaptive immune cells (B and T lymphocytes) [8]. Tumor-associated macrophages (TAMs) are derived mainly from circulating monocytes that are recruited into the tumor in response to various chemokines and growth factors produced by tumor and stromal cells.

Various signals, such as Toll-like receptor (TLR) ligands and interferon gamma (IFN-γ) promote polarization of TAMs to a M1-like proinflammatory phenotype, characterized by activation of proinflammatory genes, such as interleukin-1β and tumor necrosis factors (TNF)-α. Many factors promote polarization of TAMs to a M2-like phenotype (also termed alternatively activated), which express high levels of anti-inflammatory cytokines, scavenging receptors, angiogenic factors, and proteases that augment tumor progression by promoting angiogenesis, tumor cell invasion and metastasis, and suppress adaptive immunity [9,10]. Repolarization of TAMs to antitumor phenotypes is a potential therapeutic strategy of cancer [9].

MiRs may play a key role in tumorigenesis by promoting M2-like TAM polarization and inhibition of tumor infiltration with CD8+ cytotoxic T lymphocytes (CTLs). Deletion of the miRNA-processing enzyme DICER in macrophages stimulates M1-like TAM activation with recruitment of activated CTLs to the tumor. CTL-derived IFN-γ amplified M1 polarization of DICER1-deficient TAMs and inhibited tumor growth [11]. Overexpression of miR-511-3p in TAMs suppresses protumoral genes and inhibits tumor growth [12]. Increasing miR-511-3p activity in TAMs could be a potential therapeutic strategy to repolarize them to an antitumor phenotype. MiR-21-3p and -5p have an immunosuppressive effect by inhibiting the migration of CTLs into the tumor by decreasing the secretion of CCL-3 and CXCL-10 [13].

MiR-let-7b expression is upregulated in prostatic TAMs and promotes prostate carcinoma cell mobility and angiogenesis, while treatment with let-7b inhibitors reduce angiogenesis and cell mobility [14]. Several exosomal miRs have been shown to promote the M2-like phenotype of TAMs and accelerate tumor progression, such as miR-222-3p and miR-940 in epithelial ovarian cancer [15,16], miR-145 in colorectal cancer (CRC) cells [17], miR-103 in lung cancers [18]. Conversely, exosomal miR-21 could be transferred from M2-like TAM to gastric cancer cells, where it inhibits apoptosis of cancer cells through regulation of PI3K/Akt signaling and Bcl2 expression [19].

In contrast, miR-155 [20] and miR-19a-3p [21] expression is low in TAM and inhibits tumor growth and metastasis in a mouse breast cancer model by reprograming M2-like macrophages toward classic M1-like activation. Similarly, miR-142-3p is downregulated in TAM and inhibits glioma growth probably by promoting M2-like TAM apoptosis [22].

Cationic Bletilla Striata polysaccharide (cBSP) is a modified herb extract, which exhibits high affinity for macrophages and can be used as a nonviral drug delivery system targeting these cells [23]. The packaged let-7b into cBSP can be released in response to the tumor acidic microenvironment with the help of a pH-responsive material PEG-histamine-modified alginate [24]. This nanocomplex could reprogram TAMs towards M1-like and inhibit tumor growth in a breast cancer mouse model [24]. Otherwise, miR-18a was packed in a grapefruit-derived nanovector, which is specifically up taken by Kupffer cells, but not by other cells, after intravenous administration [25]. MiR-18a inhibits liver metastasis of colon cancer by inducing M1-like polarization. The therapeutic application of macrophage-derived miRNAs by using macrophage-specific delivery systems is a promising means.

### 2.2. The Role of CTBP1 in Cancer by Modulating microRNAs Expression

C-terminal binding protein 1 (CTBP1) is a co-repressor of many tumor suppressor genes that is activated by either NAD+ or NADH. However, affinity of CTBP1 for NADH is 100-fold higher, making it a molecular sensor of the metabolic state of the cell [26]. MetS-like disease, generated by chronic high fat diet (HFD), increases intracellular NADH and activates CTBP1. CTBP1 represses the gene expression of epithelial cell adhesion molecules, like E-cadherin, and promotes epithelial to mesenchymal transition (EMT), which contributes to cell proliferation and invasion [27]. CTBP1 upregulation and the resulting E-cadherin downregulation were correlated with the progression of human hepatocellular carcinoma (HCC). CTBP1 increased breast [28] and prostate [29] tumor growth and metastasis, in MetS mice, by modulating multiple genes and miRNAs expression implicated in the extracellular matrix (ECM), cell adhesion, and cell proliferation. Thus, CTBP1 regulates 42 miRNAs in MetS mice with breast cancer. Several miRNAs were previously reported altered in cancer, such as let-7e-3p, miR-4448, miR-223-3p, miR-3151-5p, miR-940, miR-378a-3p, miR-146a-5p, and miR-124 [28]. Some of them have been involved in breast cancer progression and metastasis, miR-378a-3p, miR-146a-5p, let-7e-3p, miR-381-5p, miR-194-5p, miR-494-3p) [30]. In prostate cancer, CTBP1 regulates a cluster of miRNAs that target cell adhesion. As prostate cancer progresses in the setting of MetS, CTBP1 increases, resulting in repression of miR-205-5p together with upregulation of oncomiRs, like: miR-19b-3p, miR-29c-3p, miR-30b-5p, miR-301a-3p, miR-454-3p [29].

Altogether, these results suggest that CTBP1 hyperactivation is critical for MetS effect on cancer progression and metastasis since CTBP1 depletion diminishes the detection of circulating tumor cells [30] and the number and size of metastasis [29].

### 2.3. The Role of Peroxisome Proliferator-Activated Receptor Gamma (PPARγ) in Obesity and Cancer

PPARγ is a transcription factor highly expressed in adipose tissue with a central role in differentiation and function of mature adipocytes. PPARγ functions as a tumor suppressor, it promotes apoptosis and inhibits cancer cell proliferation, angiogenesis, and tumor microenvironment inflammation [31,32]. Diet-induced obesity and/or IR induce a decline in the expression of PPARγ, with potential relevance in obesity-related cancers. Epigenetic regulation of PPARγ may explain its down-regulation, and several microRNAs have been implicated [33]. MiR-27b, 130b, and 138 are upregulated in obesity. MiR-27b and 130b target directly PPARγ [34,35], while miR-138 indirectly inhibits the expression of PPARγ [36]. The upregulation of miR-27b, 130b, and 138, as well as PPARγ promoter hypermethylation in obese patients was responsible for PPARγ suppression and susceptibility to CRC [37]. Similarly, microRNA-130b up-regulation promotes lung cancer progression by suppression of PPARγ, which in turn activates BCL-2 and VEGF-A [38]. Beside obesity, miR-27b may be upregulated by human papillomaviruses HPV16 E7 in cervical cancer tissue, which suppresses the expression of PPARγ and increases the level of oncogenic pH regulator, Na+/H+ exchanger isoform 1 (NHE1) [39].

Classically, PPARγ exerts an antifibrotic effect by antagonizing TGF-β signaling. However, an additional mechanism has been proposed recently. PPARγ was identified as a major transcription factor which regulates a class of 8 miRNAs with antifibrotic properties, i.e., miR-29c, miR-335, miR-338, let-7a, let-7c, let-7g, miR-30d, and miR-30e. This miRs network proved to be active in three human fibrosis-associated carcinomas: HCC, breast, and lung carcinomas [40].

### 2.4. PI3K/AKT—Common Pathway in MetS and Cancer

A common mechanism which might explain the crosstalk between MetS and cancer is the PI3K/AKT pathway which represents a direct target of several miRs and is disturbed in both conditions. Chakraborty et al. systematized the influence of a plethora of miRs acting on insulin signaling pathway such as: miR-320, miR-383, miR-181b on IGF-1/IGF1R; miR-128a, miR-96, miR-126 on IRS; miR-29, miR-384-5p, miR-1 on PI3K; miR-143, miR-145, miR-29, miR-383, miR-33a/b miR-21 on AKT/PKB; and miR-133a/b, miR-223, miR-143 on GLUT4 [41]. Additionally, miR-221 binds to PI3K mRNA and inhibits glucose uptake in HepG2 cells [42]. On the other site, the PI3K/AKT pathway represents the direct target of mir-221 in several types of cancer, like laryngeal cancer [43], pancreatic cancer [44], breast cancer [45], and prostate cancer [46].

Striking evidence of a crosstalk between adipose tissue and prostate cancer, through miRNAs, has been recently reported by Massillo et al. [46]. In their study, mice with MetS characteristics, induced by a high-fat diet, were injected with prostate cancer cells and assessed for tumor growth and miRs expression. The authors found a group of 5 miRNAs (miR-221-3p, 27a-3p, 34a-5p, 138- 5p, and 146a- 5p) that were increased in gonadal adipose tissue, prostate tumors, and plasma of MetS mice compared to control animals. From these, miR-221-3p, 27a-3p, 34a-5p, and 146a-5p were confirmed to be important in prostate cancer patients [46].

MiR-221-3p proved again to mediate the crosstalk between adipose tissue and tumor growth in breast cancer [47]. The overexpression of mir-221-3p in human adipocytes impairs adipocyte lipid storage and differentiation, while conditioned medium obtained from miR-221-3p overexpressing adipocytes increased the invasion and proliferation of MCF-7 cells. Of great interest is the fact that the expression of mir-221-3p in subjects undergoing mastectomy, in the adipose tissue adjacent to BC, increases with the grade of BC. A negative correlation between the overexpression of mir-221-3p and AdipoQ was noticed, thus the inhibitory effects on BC growth of AdipoQ were lost [47].

Additionally, the PIK/AKT signaling pathway represents a target for mir-145. The upregulation of mir-145 in obese mice prevents insulin-stimulated AKT activation [41]. The overexpression of mir-145 in CRC leads to the inhibition of the PI3K/AKT signaling pathway, which in turn increases the sensitivity CRC to oxaliplatin [48]

A recent multi-omics approach and computational analysis on human visceral adipocytes compared the dysregulated miRNAs in obese subjects with or without CRC with normal weight controls [49]. MiR-193b-3p, miR- 125a-5p, and miR-1247-5p, were found to be downregulated in both cancer and obese conditions. Both miR-193b-3p and miR-1247-5p act as tumor suppressors in different types of cancer, suggesting that their repression in adipose tissue from obese and CRC individuals could have a potential tumorigenic role. Several pathways were dysregulated in both obesity and CRC networks: inflammatory signaling, such as IL-37 and IL-13, TGF-beta signaling, PTEN regulation, type I IFN signaling, SUMOylation, RNA metabolism, pathways related to vesicle budding and endocytosis [49].

### 2.5. Caveolin-1 (CAV1) Signaling

CAV1 is the main component of caveolae which are small invaginations at the plasma membrane, especially in endothelial cells and adipocytes. They are involved in endocytic and exocytic pathways as well as signal transduction.

CAV1 a critical regulator of the insulin receptor and insulin signaling, by stabilizing caveolae and their associated insulin receptors [50]. CAV1 is targeted by several miRs, which are upregulated in obesity and IR, like miR-103/107 [50] and miR-221/222 [51]. Upregulation of CAV-1 upon miR-103/107 or miR-221/222 inactivation improves insulin sensitivity and decreases glucose levels [50,51]. Interestingly, the expression on miR-221/222 correlated with BMI and HOMA-IR in postmenopausal women, with DM2 and/or breast cancer, but the highest serum levels were found in women with both diseases [51].

Downregulation of CAV1 in breast cancer promotes tumor relapse, drug resistance, and poor outcome [52,53], and has been related to the increased expression of several growth factors and regulators, like stromal cell-derived factor-1 (SDF-1), epidermal growth factor (EGF), and fibroblast-specific protein-1 (FSP-1) [54].

It has been shown that hyperglycemia induces epithelial to mesenchymal transition (EMT), a key process in metastatic disease, in MCF-7 and T47D human breast cancer cells. By blocking the hyperglycemia-induced EMT phenotype, cell growth was suppressed but invasive capacity increased through a CAV-1 dependent mechanism [55]. Similarly, diet-induced obesity increased melanoma progression in male C57BL/6J mice by enhancing Cav-1 and FASN expression in tumors [56].

### 2.6. Wnt/β-catenin Signaling 

*The canonical Wnt/β-cate pathway* is involved both in cancer and in various metabolic processes including adipogenesis and glucose homeostasis [57]. Dysregulation of the Wnt/β-catenin pathway is one of the most relevant driving forces in cancer development and metastasis. Several miRs have been implicated in different types of cancer, like: CRC, breast cancer, ovarian, prostate cancer, through Wnt/β-catenin pathway dysregulation [58].

The mechanism of increased risk of HCC in obese individuals was recently elucidated [59]. It has been demonstrated that infiltrating macrophages induced by liver steatosis promotes growth of tumor progenitor cells through Wnt/β-catenin activation. Indeed, activation of Wnt signaling predicts 90% of tumors in a large cohort of patient samples [59]. Moreover, the obesity-related upregulation of miR-27a has been shown to promote metastasis of HCC through activated Wnt/β-catenin signaling [60].

### 2.7. PTEN Signaling

The tumor suppressor phosphatase and tensin homolog deleted on chromosome 10 (PTEN) is a lipid phosphatase with role in obesity and IR [61] and several cancers [62]. PTEN is a potent tumor suppressor, and its loss of function is frequently encountered in cancer. Even a slight decrease in PTEN levels and activity results in cancer susceptibility or tumor progression.

Mostly through the activation of the PI3K/AKT/mTOR pathway, PTEN deficiency influences a variety of biologic processes that sustain cancer cell growth and proliferation [63]. Because metabolic effects of insulin are mediated through the same pathway, PTEN has a critical role in modulating insulin-induced glucose uptake and insulin-induced suppression of hepatic gluconeogenesis. Several miRs have been shown to inhibit the expression of PTEN at the post-transcriptional level in a variety of cancer types, like miR-21, miR-22, miR-214, mir-17–92, mir-106b-25, mir-367–302b, and mir-221–222 [64].

Interestingly, it has been shown that astrocyte-derived exosomes, containing anti-PTEN microRNAs, suppress PTEN expression in the brain metastatic tumor cells [65]. Recently, polyphenols proved to have chemopreventive potential in obesity-induced prostate cancer by rescuing PTEN expression [66].

PTEN interacts and facilitates TGF-β impacts on cell proliferation. Up regulation of PTEN diminishes TGF-β-mediated AKT phosphorylation, precluding the functions of TGFβ on cell proliferation while its down-regulation promotes TGF-β effects on induction of the PI3K pathway.

### 2.8. The miRNA-Processing Enzyme DICER

DICER has a central role in the final steps of the miRs processing pathway. It has been involved in both pancreas function and insulin signaling [67]. More recently, it has been shown that adipose tissue is an important source of circulating exosomal miRs, which can regulate gene expression in distant tissues. Adipose-tissue-specific knockout of DICER in mice, as well as humans with lipodystrophy, significantly decreased the circulating levels of exosomal miRs [68]. 

Upon deletion of DICER, several molecules and receptor tyrosine kinases, which are involved in IR and cancer development, were hyperphosphorylated (IGF1, IR, IRS-2, and STAT3) [69].

Many studies revealed that DICER acts as a tumor suppressor and loss of DICER protein expression in invasive tumor samples. Low DICER expression was associated with poor prognosis in ovarian cancer (HR = 1.93), otorhinolaryngological tumors (HR = 2.39), hematological cancers (HR = 2.45), and neuroblastoma (HR = 4.03) [70]. Several miRs have been shown to target and repress DICER, like: miR-103/miR-107 in breast cancer [71], miR-221/miR-222 and miR-29a in triple negative breast cancers [72]. Similarly, miR-103 target and suppressed DICER and PTEN, promoting proliferation and migration of CRC [73].

In contrast, transcriptional activation of DICER through ERK/Sp1 activation causes pancreatic cancer progression and resistance to gemcitabine [74].

### 2.9. PPARα -FOXP4- NOTCH Pathway

MiRs has been shown to regulate PPARα, a known regulator of both adipogenesis and carcinogenesis, suggesting that miRNAs play a vital role between obesity and cancer [75].

PPARα was demonstrated to act as an oncogene, especially in breast cancer. Thus, Chen et al. showed that ectopic expression of PPARα increases cell proliferation of breast and pancreatic cancer cells via a novel target gene, *carnitine palmitoyl transferase 1C* (*CPT1C*) [76]. Interestingly, coculture of breast cancer cells with mature adipocytes increased secretion of proinflammatory cytokines and chemokines and consequently the proliferation, migration, and invasive of cancer cells. The most deregulated miRs were miR-3184-5p (upregulated) and miR-181c-3p (downregulated) and they target directly FOXP4 and PPARα, respectively [77]. These data strongly proved that adipocytes-secreted factors drive the breast cancer progression and metastasis in obese patients. FOXP4 is a family of forkhead box transcription factors, with a critical role in cancer growth and metastasis in different types of cancer. Likewise, FOXP4 expression was negatively regulated by miR-3184-5p in NSCLC [78].

The Notch signaling pathway is a highly conserved pathway, which is required for cell–cell communication and many biological processes such as proliferation, organ development, differentiation, metabolism, and maintenance of stem cells [79]. The activation of the Notch pathway is associated with poor patient survival in breast cancer [80]. More interestingly, a recent study demonstrated that activation of Notch induces dedifferentiation of mature adipocytes and promotes tumorigenic transformation in mice [81]. Battle et al. [82] demonstrated the role of obesity-induced leptin-Notch signaling in breast cancer. These studies support the concept that adipocytes activate in the breast tumor microenvironment, the Notch-EMT signaling, increasing the migration and invasion, which in turn, promote a more aggressive metastatic tumor.

### 2.10. SRC/SOX2/c-MYC Pathway

It has been shown recently that co-culture of breast cancer cells with immature adipocytes or cytokines upregulates miR-302b via activation of SRC. Picon-Ruiz et al. [83] showed that the SRC action sustains cytokine induction and promotes SOX2-dependent miR-302b upregulation, to further induce MYC and SOX2 expression and increase malignant stem cells. Feed-forward mechanisms established by SRC-driven SOX2 and miR-302b induction would sustain subsequent cytokine production after initial exposures.

SRC was shown to activate NF-kB via STAT3 to induce IL6 and oppose Let7-mediated IL6 repression [84]. SOX2 is amplified in certain cancers and can drive clonogenic tumor growth [85]. Moreover, miR-302b expression in breast cancers was associated with expression of stem cell markers, nodal metastasis, and poor patient outcome [86].

**MiR-21**

MiR-21 levels were found to be increased in patients with type 1 (T1D) and type 2 diabetes (T2D), the circulating levels of miR-21 reflecting the degree of pancreatic inflammation [87]. Contrarily, there are studies that did not find discrepancies in the miR-21 serum levels in patients with or without diabetes [88]. Interestingly, miR-21 antagomir ameliorates metabolic disturbances in T2D patients by up-regulating the expression of the target gene *TIMP3* [89]. Furthermore, the inhibition of the miR-21 expression may represent a key point in the improvement of glycemic control through PPARc and GLUT4 regulation [90]. The expression of mir-21 is reduced in insulin-resistant adipocytes. Additionally, the over-expression of mir-21 improved glucose metabolism and insulinemia via the PTEN/PI3K/Akt pathway [91].

Low levels of miR-21 were identified in the peripheral blood mononuclear cells of obese, regardless of the presence of diabetes, which was negatively correlated with inflammatory cytokine production [92]. By targeting 3′UTR of STAT3 mRNA, miR-21 regulates the adipose cell proliferation and differentiation [93]. Additionally, the anti-miR-21 proved to be efficient for miR-21 inhibition which consequently led to weight loss via its target genes: transforming growth factor beta receptor 2 (TGFRB2), PTEN, and Sprouty1 and 2 [94]. In subjects with MetS, the circulatory levels of mir-21 were decreased compared to the patients without MetS [95].

Dysregulation in the expression miR-21 was also observed in neoplasms, miR-21 being one of the most studied oncomirs (see Table 1). Thus, its overexpression promotes metastatic phenotype of cancers by targeting RHOB and suppressing its activity [96].

MiR-21 is a determinant of prostate cancer aggressiveness by targeting 3′-UTRs of PDCD4 and maspin. Upregulation of mir-21 was mediated by NADPH oxidase system-ROS generation—Akt pathway [97]. Similarly, increased levels of mir-21 were associated with the prostate cancer aggressiveness, thus being useful in the identification of high-risk patients [98]. MiR-21 emerged as an independent risk factor for recurrence of prostate neoplasia in patients with obesity but not in non-obese subjects [99].

As mentioned above, miR-21 exerts its oncogenic effect on HHC cells by regulating the protein kinase B (AKT)/extracellular signal-regulated kinase (ERK) pathways [100].

As other miRs, mir-21 is overexpressed in osteosarcoma tissue playing a key role in tumor invasion and migration, having the tumor suppressor gene, RECK, as a target in the aforementioned process [101]. MiR-21 implication in CRC was intensively studied. The interplay between CASC7, mir-21, and IGN3 might have a key role in CRC progression [102]. The long intergenic noncoding RNA, LINC00312 disrupts the proliferation, migration, and invasion of CRC cells by reducing the expression of miR-21 and consequently increasing PTEN expression [103]. MiR-21 promotes the development of CRC by targeting directly RHOB, thus promoting the cancer cells proliferation, invasion, and inhibiting the programmed cell death [104]. Additionally, miR-21 promotes the CRC through the downregulation of Sec23A [105]. The inhibition of miR-21 and the consequent increased expression of *TIMP*-3 and *RECK* proved to reduce the aggressiveness and the ability to metastasize [250].

The increased expression of oncomir-21 was identified in breast cancer, being correlated with circulating hormone levels. It plays an important role in tumor progression and aggressiveness by targeting STAT3 [106] and PI3KR1 [107].

The overexpression of miR-21 in non-small cell lung cancer (NSCLC) was associated with enhanced tumor aggressiveness and invasiveness through PTEN deregulation [108].

The implication of miR-21 in the pathogenesis of papillary thyroid carcinoma is mediated by the VHL/PI3K/AKT pathway, thus increasing the aggressiveness of this type of cancer. In addition, VHL may be a valuable tool in order to counteract the effects exerted by miR-21 [109].

Gastric cancer progression is promoted by the overexpression of miR-21 which regulates the expression of tumor suppressor genes *PTEN* and *PDCD4* and consequently increases the cancers’ aggressiveness [110]. The inhibition of miR-21 in gastric cancer proved to be a promising strategy for counteracting its biological effects by reducing tumor aggression [251].

A potential mechanism that explains the aggressiveness of melanoma is the overexpression of oncomiR-21 that inhibits TIMP3 which regulates the matrix metalloproteinases activity. Therefore, the inhibition of miR-21 may be promising in melanoma management [111].

MiR-21/Sox2/β-catenin is one of the pathways implicated in glioma pathogenesis. Sox2 overexpression not only increased the expression of β-catenin but also enhanced tumor aggressiveness [112]. Additionally, miR-21 also plays a prognostic role in glioma patients [209]. The miR-21 is also correlated with the histological grade of gliomas and its expression is modulated by the STAT3/β-catenin pathway. Furthermore, the tumors’ invasion capacities are augmented as a result of the regulatory effect of miR-21 over RECK [113].

MiR-21 is implicated in the pathogenesis of oral squamous cell carcinoma (OSCC) through its target gene, *PTEN*. Additionally, the expression of miR-21 and *PTEN* were associated with the tumors’ grade [114]. Tongue squamous cell carcinoma oncomir-21 increased tumors’ aggressiveness by inhibiting DKK and consequently activating the Wnt/β-Catenin pathway [115]. As in OSCC, the miR-21/PTEN pathway is implicated in cervical cancer pathogenesis [117].

The implication of miR-21 in the progression of renal cell cancer (RCC) was also established [118]. Regarding nasopharyngeal carcinoma, miR-21 expression proved to be up-regulated in advanced stages and was also correlated with the presence of metastatic adenopathy. Additionally, miR-21 seems to regulate the expression of BCL2 protein by targeting directly the BCL2 mRNA [116].

The aforementioned findings shed some light on the implication of miR-21 and its pathogenic pathways implicated in both MetS and cancer. Thus, miR-21 PTEN-induced deregulation is implicated in CRC [103], NSCLC [108], gastric cancer [110], OSCC [114], and cervical cancer [117], being closely correlated with the tumor aggressiveness [110], grade [114], and invasiveness [108]. Additionally, the miR-21/PTEN/PI3K/Akt pathway is implicated in the glucose metabolism and insulin homeostasis [91]. STAT3 represents a common target for miR-21 which is implicated in both breast cancer pathogenesis [106] and adipose cell proliferation and differentiation [93]. While the inhibition of miR-21 leads to an increased expression of TIMP-3 and a consequent reduction in the CRC aggressiveness [250], the overexpression of miR-21 inhibits TIMP-3 in melanoma [111]. The up-regulation of TIMP-3 improves the metabolic imbalances in T2D subjects [89]. Thus, PTEN/PI3K/Akt, STAT3, and TIMP-3 represent major pathways that mediate the crosstalk between MetS and cancer.

**MiR-24-3p**

MiR-24-3p is down-regulated in T2D and MetS subjects. MiRs levels correlated with serum insulin and HbA1c levels in individuals with T2D or MetS, and with higher BMI, dyslipidemia, and family history [252]. T2D patients expressed low levels of miR-24-3p [253]. Obesity induces overexpression of miR-24-3p which in turn suppresses HDL uptake, lipid metabolism, and steroid hormone intake by inhibiting Scavenger Receptor B-1 (SRB1) [254].

MiR-24-3p functions as a tumor suppressor. Overexpression of miR-24-3p inhibits p27Kip1 [122] and Bim expression, therefore increasing growth and proliferation of breast cancer [123].

In CRC, miR-24-3p has a tumor suppressor function, down-regulation of miR-24-3p promotes CRC development and progression and plays a potential role in prognosis and therapy. Overexpression of miR-24-3p inhibited cell proliferation, migration, and invasion, indicating a key role for dysregulation of miR-24-3p in CRC tumorigenesis, and might have a therapeutic potential to suppress CRC progression [120].

**MiR-26a**

MiR-26a is involved in insulin signaling pathways through its action on GSK3β, PKCδ, and PKCθ, in fatty acid metabolism and gluconeogenesis through its effect on the genes that regulate PCK1 and TCF7L2 expression. It was demonstrated in animal models that a slight decrease in miR-26a contributes to the development of IR and T2D, and that a slight increase can prevent the development of complications associated with obesity [255]. At the same time, it was proved that miR-26a is a potent inhibitor of adipocyte differentiation, inhibiting adipogenesis through its suppressive action on Fxl19 [256].

MiR-26a plays a dual role in tumorigenesis, functioning both as a tumor suppressor and as an oncomir. Studies have shown that miR-26a acts as a tumor suppressor in lung cancer, breast cancer, HCC, rhabdomyosarcoma, prostate cancer, melanoma, papillary thyroid carcinoma, gastrointestinal carcinomas through its action on key proteins involved in the control of cell proliferation such as p53, SMAD1, EZH2, IL-6-Stat3, CTDSP1/2/L, SODD, CKS2, FGF9. On the other hand, miR-26a is involved in tumor pathogenesis through its oncogenic effect. For example, in glioma, miR-26a acts as a tumor promoter through its action on the PTEN and PHB signaling pathways, in ovarian cancer through effect on ERα, and in cholangiocarcinoma by reducing GSK3 [124].

MiR-26 was involved in triple-negative breast cancer development by down-regulating the expression of BRCA1 [126]. Additionally, in breast cancer, miR-26 expression was correlated with tumor size, HER2 status, and ki-67 value [125].

An interesting clinical implication could be the use of miR-26a for therapeutic purposes, a number of studies demonstrating that it can influence sensitivity to conventional chemotherapy [124]. For example, in pancreatic cancer, miR-26a overexpression facilitates local accumulation and sensitivity to gemcitabine [257].

**Mir-26b**

MiR-26b is among the obesity-related miRNAs, being reduced in adipocytes from visceral obesity and IR. The expression level of miR-26b negatively correlates with increasing BMI and IR in human obese subjects [258]. MiR-26b promotes Glut4 translocation to the plasma membrane and insulin-stimulated glucose uptake in human mature adipocytes. MiR-26b increases insulin sensitivity via the PTEN/PI3K/AKT pathway [127].

The MiR-26 family of microRNAs (miR-26a-1, miR-26a-2, and miR-26b) was proved to be a major inhibitor of adipogenesis, at least in part, by repressing expression of Fbxl19 [116]. Deletion of all miR-26 in mice resulted in excess body fat and dyslipidemia in animals fed normal chow due to precocious differentiation of adipocyte progenitor cells [126].

MiR-26b was shown to be down regulated and exhibited an antitumor effect in glioma and lung cancer cells by targeting cyclooxygenase (COX)-2 [127,128], and in HCC by targeting EphA2 [129]. Overexpression of miR-26b inhibited proliferation, invasion, and migration of cancer cells and might be a therapeutic strategy in these tumors [127,129].

**MiR-27**

MiR-27 has shown to be overexpressed in obesity due to hypoxic condition. The miR-27 family inhibits PPAR-γ function, activates Wnt1 signaling [259], and suppresses GLUT-4 and PI3K signaling, leading to hyperglycemia, IR, and hyperlipidemia [34].

The MiR-27 family was upregulated in multipotent stem cells isolated from omentum of morbidly obese patient compared to lean subjects, leading to a dysregulation of important pathways implicated in the early stages of adipocyte differentiation such as Wnt, TGFβ/Smad, and PPARγ/C/EBPα pathways [260]. Kang et al. described MiR-27 (miR-27a and miR-27b) as an anti-adipogenic microRNA by targeting prohibitin (PHB) and impairing mitochondrial function, which leads to a reduction of adipogenesis [131].

MiR-27 acts as a tumor suppressor in breast cancer, by targeting SPRY1, BAK, FOXO1, and CBLB/GRB2 [132,261,262]. Upregulation of miRNA-27 was correlated with a higher risk of gastric cancer, by promoting transformation of cancer-associated fibroblasts [130].

**MiR-27b**

MiR-27b was shown to be upregulated in different models of IR [131]. miR-27b directly suppresses the expression of insulin receptor (INSR) by targeting 3’UTR of INSR. Modulation of miR-27b expression in a HFD-induced IR mice model improved glucose tolerance and insulin sensitivity in adipose tissue by increasing the expression of its target gene INSR [131].

The upregulation of miR- 27b in obese patients was associated with susceptibility to CRC through PPARγ promoter hypermethylation and downregulation [37].

MiR-27b was upregulated in cervical cancer cells and tissues [263]. It acts as an oncogene with a role in the progression of cervical cancer. Upregulation of miR-27b increased proliferation, cell cycle transition from G1 to S phase, migration, and invasion of C33A cells, by modulating cadherin 11 (CDH11) and EMT [133]. Recently, it has been shown that miR-27b may be upregulated by human papillomaviruses HPV16 E7, which suppresses the expression of PPARγ and increases the level of Na+/H+ exchanger isoform 1 (NHE1) [39].

Contrary, MiR-27b has been reported as a tumor suppressor in several cancers. MiR-27b was decreased in both NSCLC tissues and cell lines, while its overexpression suppressed NSCLC cells proliferation and invasion, by targeting LIM kinase 1 (LIMK1) [264] and Sp1 [134].

**Mir-30**

The miRs expression analysis in ATMs of high fat diet (HFD)-induced obesity in mice compared to lean normal chow diet mice revealed substantial dysregulations of miR-30 which led to a M1 polarization of ATM in the HFD mice. The inhibition of miR-30 proved to induce inflammation through the DLL4-Notch signaling-pathway, thus the anti-inflammatory role of miR-30 in macrophages [265].

Aside from obesity and inflammation, miR-30 is also implicated in breast cancer [135], NSCLC [136], and pancreatic cancer [137]. In breast cancer cells, miR-30 was the highest expressed miRs and its expression was much higher in invasive tumor cells than in mass tumor cells. Additionally, silencing miR-30 reduced the invasion and growth abilities of cancer cells but at the same time prolonged their survival time [135].

In NSCLC, the levels of miR-30 are down-regulated which leads to the overexpression of MMP19 in pulmonary cancer. MMP19 is implicated in the promotion of tumor genesis and metastasis so it can be a predictor of a poor prognosis in lung cancer patients. Explanatory for the prognostic implications of miR-30/MMP19 pathway is the effect of MMP19 on EMT that results in loss of intercellular adhesion. Furthermore, MMP19 is responsible for high expression levels of proliferative growth factors [136].

In pancreatic cancer, miR-30 was implicated in gemcitabine treatment resistant cancer lines and increased invasiveness but had no impact on cell proliferation [137].

**MiR-31**

MiR-31 has a higher expression in visceral adipose tissue of obese and diabetic patients compared to healthy subjects, but it is not clear yet if this is the cause or the consequence of obesity or T2D. The target genes are involved in adipogenesis (*PPARg*, *PRKAA1*, and *ACACA*) and insulin signaling (*GLUT4* and *IRS1*) [266]. The secretion of miR-31 from adipose tissue-derived stem cells promote angiogenesis by targeting the factor inhibiting HIF-1a, indicating a possible correlation between dysfunction of adipose tissue and tumorigenesis [267]. The expression of miR-31 in tumor tissue of patients with head and neck squamous cell carcinoma (HNSCC) was higher than that in adjacent normal tissues. Highly expressed MiR-31 was associated with tumor differentiation, metastasis, and poor prognosis. The expression of miR-31 significantly reduced the expression of the tumor suppressor gene, *adenine thymine-rich interactive domain 1A* (*ARIDIA*) [138]. Similarly, higher miR-31 expression was detected in rectal cancer tissues compared with adjacent tissues [139]. In vitro overexpression of miR-31 increases invasiveness, while the proliferation and invasion of rectal cancer cells were inhibited by inhibiting the expression of miR- 31 [139]. Similarly, increased miR-31 expression in CRC tissue was associated with disease invasiveness and poor prognosis, by targeting factor-inhibiting HIF-1α (FIH-1) [140].

MiR-31 proved to have a dual effect on breast cancer. It increases the primary tumor growth but most important, it is key anti-metastatic miRs in breast cancer. MiR-31 expression was four-fold reduced in a non-metastatic breast cancer cell line (MCF-7), while its expression in a metastatic breast cancer line (MDA-MB231) was decreased 100-fold [141]. MiR-31 targets RhoA mRNA, which is implicated in cell movement and cytoskeleton. Another study confirmed that miR-31 expression is lost during breast cancer progression [268]. MiR-31 directly binds to the 3′-UTR of G protein alpha-13 (GNA13) and suppresses its activity. GNA13 is most highly expressed in more aggressive breast cancer cells [268].

Similarly, the pro-tumorigenic effect of miR-31 loss has been described in malignant mesothelioma (MM) of pleura [142]. MM cell lines derived from patients with aggressive tumors did not express miR-31 due to homozygous deletion of the miR-31-encoding gene that resides in 9p21.3. Re-introduction of miR-31 inhibited proliferation, migration, invasion, and clonogenicity of MM cells. The pro-survival phosphatase PPP6C possesses three potential binding sites for miR-31 in its 3′-UTR and was down-regulated by miR-31 introduction and up-regulated in clinical MM specimens [142].

MiR-31 demonstrated a tumor suppressive role in glioma tumor. Its expression was markedly reduced both in glioma cell lines and in glioma tumor specimens compared with the adjacent human brain tissues [143]. miR-31 downregulation was due to hypermethylation of its promoter region. It modulates Dock1 expression, while Dock1 promotes the IL8-induced chemotaxis and mesenchymal transition of glioma cells through the NF-jB/Snail signaling pathway. Moreover, the therapy with a DNA methyltransferase inhibitor restored miR-31 expression in glioma cells and inhibited cell invasion. Similarly, miR-31 is downregulated and acts as a tumor suppressor in gastric [269] and serous ovarian cancer cell lines as well in serous ovarian tumors via regulation of zeste homolog 2 (ZH2) [144]. Overexpression of miR-31 causes cell cycle arrest, inhibition of proliferation, migration, and invasion of the gastric, ovarian, osteosarcoma, and prostate cancer cell lines [144,269].

**MiR-34a**

Overexpression of miR-34a in visceral fat of overweight/obese subjects is associated with IR and metabolic inflammation. Lipid loaded mature adipocyte-secreted exosomes transport miR-34a to macrophages and suppress the anti-inflammatory M2 phenotype by repressing Kruppel-like factor 4 (Klf-4) [270]. MiR-34a is a key mediator in exacerbating obesity-related systemic inflammation and metabolic dysregulation [270]. MiR-34 was also described to be increased in humans with non-alcoholic fatty liver disease (NAFLD) and T2D [271,272], and some experimental evidence indicates that increased miR-34a levels in the liver are associated with metabolic alterations [273]. MiR-34 suppresses WAT browning in obesity, by targeting Fibroblast Growth Factor Receptor 19 (FGFR19) and SIRT1 (sirtuin 1). SIRT1 suppression is linked to impaired transcriptional regulation of brown and beige markers through deacetylation of PPARGC1-α [274].

Many studies reported that miR-34a is down-regulated and acts as a tumor suppressor in human breast tissue [157]. MiR-34a acts as a tumor suppressor miR by down-regulating its target genes such as *BCL*-2 and *SIRT1* [145] and *Notch1* [146], Wnt/β-catenin signaling pathway [147], *fra*-1 [148] and *MYC* [149].

In CRC, Siemens et al. showed that the formation of distant metastases is associated with epigenetic silencing of miR-34a in primary tumors. In addition, they found that patients who subsequently developed distant metastases had a preferential up-regulation of the miR-34a targets *Snail*, c-*Met*, and β-*catenin*, which have prometastatic functions, in the primary tumors. Notably, the authors indicate that the detection of enhanced c-Met and β-catenin expression alongside miR-34a CpG methylation can have prognostic value [152].

Fujita et al. studied the role of miR-34a in anticancer drug resistance in prostate cancer cell lines. They found a significantly reduced expression of miR-34a in p53-null PC3 cells and p53-mutated DU145 cells compared to wild-type p53 LNCaP cells. Ectopic expression of miR-34a can decrease SIRT1 mRNA and protein levels, lead to cell cycle arrest and growth inhibition, and mitigate chemoresistance to the anticancer drug camptothecin by inducing apoptosis [275].

**MiR-96**

MiR-96 targets directly the 3’UTRs of INSR and IRS-1, and decreases their expression at the post-transcriptional level. Induction of miR-96 by dietary saturated fatty acids impairs insulin signaling and exacerbates hepatic insulin resistance through the suppression of INSR and IRS-1 [276]. Grape seed proanthocyanidins extracts significantly decreases HFD-induced miR-96 upregulation in mice, and reduces the expressions of miR-96 downstream molecules, FOXO1, p-mTOR, mTOR, and LC3A/B [277].

MiR-96 functions as an oncogene in several types of cancers. Thus, miR-96 is overexpressed in HCC and promotes cell proliferation, migration, and invasion by inhibiting the tumor suppressors SOX6 [159] and EphrinA5 [160]. It is upregulated in breast cancer and enhances tumor growth and progression by silencing the protein tyrosine phosphatase, *PTPN9* [161] and the tumor suppressor genes *FOXO1* [162] and *FOXO3a* [163]. PTPN9 may contribute to tumor suppression by dephosphorylation and silencing of EGFR, ErbB2, and STAT3 in breast cancer [161]. Similarly, miR-96 is significantly upregulated in NSCLC and functions as an onco-miRNA via targeting FOXO3 [164]. MiR-96 up-regulation was demonstrated in esophageal cancer, where it promotes proliferation and chemo- or radio resistance by RECK down-regulation [165]. 

Contrary, miR-96 may act as a tumor suppressor, inhibiting migration, invasion, and proliferation of glioblastoma multiforme cells via down regulation of astrocyte elevated gene-1 (AEG-1) at the mRNA and protein levels [166]. It suppresses renal cell carcinoma invasion by downregulation of Ezrin expression [167]. Similarly, Yu et al. showed that miR-96 functions as a tumor-suppressor in pancreatic cancer cells, where it decreased cell migration and invasion and decreased tumor growth via downregulation of *KRAS* oncogene [168]. More recently, it has been shown that lncRNA TP53TG1 is upregulated in pancreatic ductal adenocarcinoma and contributes to the tumor growth and progression. TP53TG1 operates as a sponge for miR-96 to weaken the suppressive effect of miR-96 on KRAS, and thus increases the expression of KRAS [278].

**MiR-100**

It has been demonstrated that normoglycemic and T2D obese patients have a reduced miR-100 expression. Obese patients with T2D show a much more reduced expression compared to normoglycemic obese patients, particularly in visceral adipose tissue compared to subcutaneous tissue. These low values are correlated with high values of TGL, basal glycemia and hsCRP. Low miR-100 induces an increase in VLDLR and differentiation of preadipocytes into mature adipocytes capable of accumulating higher lipid amounts, thus contributing to the pathogenesis of obesity.

The mTOR and IGFR signaling pathways represent direct targets of miR-100, being involved in adipogenesis, IR, as well as carcinogenesis. Inhibition of mTOR by rapamycin inhibits adipogenesis through its effect on PPAR-γ activity. IGFR mediates similar metabolic effects regarding glucose influx and IR adipogenesis. Because of structural similarities, IGFR and IR can form hybrid IR receptors [279].

Regarding carcinogenesis, miR-100 overexpression inhibits the transcription of a number of proteins in the IGF/mTOR signaling pathway, such as IGF1R, IGF2, MCL1, with a role in tumor proliferation and survival and involved in the development of several types of cancers: urinary bladder urothelial carcinoma, chondrosarcoma, endometrioid endometrial carcinoma, breast carcinoma, esophageal squamous cell carcinoma (ESCC), acute leukemia, pancreatic adenocarcinoma, H&N cancers, HCC, prostatic adenocarcinoma, NSCLC [280].

In addition to its pathogenic role, miR-100 also has a potential diagnostic, prognostic, and therapeutic role. For example, in urinary bladder cancer, studies have demonstrated that low miR-100 values represent an independent negative prognostic factor that is correlated with shorter PFS (progression-free survival) and OS (overall survival), and might be a useful instrument in patient stratification [169]. Similarly, the negative prognostic role of low miR-100 expression was demonstrated for HCC, RCC, bladder cancer, NSCLC and epithelial ovarian cancer [170], endometrioid endometrial carcinoma [171], CRC [172].

**MiR-125b**

A study showed that hepatic miR-125b expression is much higher in persons with T2DM compared to healthy individuals, and this contributes to the development of insulin resistance. The inhibition of endogenous miR-125b contributes to increasing insulin sensitivity in insulin resistance conditions. The authors also demonstrated that this effect is the result of the action of miR-125b on PI3K, which determines a decrease of insulin-induced AKT phosphorylation in hepatocytes [281].

In contrast, a study on animals showed that miR-125b expression is lower in the pancreatic cells of mice with T2D, but these presented a higher expression of DACT1, JNK, and c-Jun, demonstrating that miR-125b stimulates the increase of insulin sensitivity and pancreatic beta cell function through the inhibition of the JNK signaling pathway due to the suppressive effect on DACT1 [282].

SIRTs represent a group of enzymes with an important role in cell metabolism, inflammation, reactive oxygen species (ROS) production, and in the balance between apoptosis, survival, and cell proliferation. It was demonstrated that SIRT1 values are lower in the adipose tissue of obese persons compared to normal weight subjects, and that they are negatively correlated with miR-125b expression. SIRT1 stimulates lipolysis through its action on FOXO1 and suppresses the expression of some pro-inflammatory genes in the adipocytes and macrophages present in adipose tissue by interference with the NF-κB signaling pathway, which increases insulin sensitivity [283].

In cancer, miR-125b has an oncogenic effect in hematological cancers and a tumor suppressive effect in solid cancers. For example, in diffuse large B-cell lymphoma (DLBCL), constitutive activation of the NF-κB signaling pathway occurs through the suppressive effect of TNFAIP3 which normally inhibits the activation of this pathway by miR-125b [173]. Other targets of miR-125 involved in the development of hematological cancers are MAPK11, IRF4, and the TET2-VEGFA pathway in acute leukemia [174]. Tumor suppressive effects were observed in HCC, CRC, RCC, salivary gland carcinoma, thyroid cancer, laryngeal carcinoma, osteosarcoma, prostatic adenocarcinoma, melanoma, Ewing sarcoma, glioblastoma, gallbladder cancer through its action on mRNA genes, with effects on many pathways involved in carcinogenesis such as Wnt, PI3K/Akt, STAT-3, MAPK, NF-κB, p53 [175].

Overexpression of miR-125b was associated with resistance to cetuximab treatment in CRC and SCCHN, while in other cancers such as NSCLC, HCC, breast cancer, uterine cervical cancer, its overexpression increased sensitivity to chemotherapy. MiR-125b was also proposed as a potential marker of response to immunotherapy in NSCLC [284].

Mir-125b is implicated in the crosstalk between IR/MetS and cancer via its direct action on the PI3K/Akt pathway. Mir-125b decreases insulin sensitivity and disrupts glucose homeostasis by targeting PI3K in liver cells [281]. Additionally, mir-125 and its direct targets ErbB2, ErbB3 are implicated in prostate cancer initiation and progression through the PI3K/Akt pathway [285]

**MiR-126**

One of the targets of miR-126 is IRS-1, a protein with an important role in signal transduction in the insulin signaling pathway. It was demonstrated that low miR-126 expression is involved in the development of IR through the increase in the expression of these proteins [41]. Low values of miR-126 are also found in subjects with prediabetes compared to healthy individuals, a study even demonstrating the correlation of its serum values with the risk for subsequent T2D [286]. Another mechanism by which miR-126 is involved in the pathogenesis of inflammation and IR is through its effect on CCL-2 release from human adipocytes and macrophages [287].

In addition to the regulatory effect on the insulin signaling pathway [41], the miR-126/IRS-1 axis is involved in cancer pathogenesis [176]. IRS-1 disruptive effect on cell growth and DNA repair and replication might explain the mir-126 implication in cancer development and progression. MiR-126 acts as a tumor suppressor and a low expression has been observed in many cancers–carcinomas of the GI tract, lung, breast, prostate, thyroid. Downregulation of this miR facilitates tumor progression, migration, angiogenesis, and survival through its action on several genes and molecular pathways involved in oncogenesis such as *PI3K, KRAS, EGFL7, CRK, ADAM9, HOXA9, IRS-1, SOX-2, CADM1, PAX4, SLC7A5,* and *VEGF* [176].

**Mir-130**

Among white adipose tissue deregulated miRs, of great importance is miR-130 which might be overexpressed in the context of inflammatory stimulation, by TNF-alpha, leading to adipose cell dysfunction [177]. In diet induced obesity, mir-130 inhibition of APCDD1 leads to defective adipose cell differentiation through a plethora of laborious pathways [288]. Adolescents suffering from obesity proved to have higher plasmatic levels of mir-130 [289].

In gastric cancer cells, mir-130 enhanced their proliferation and invasion abilities. By targeting miR-130 with MRPL39, the aforementioned effects were counteracted through an anti-tumor effect [290].

**Mir-143**

Upregulation of mir-143 in dietary mouse models of obesity impairs insulin-stimulated AKT activation, through downregulation of oxysterol-binding protein-related protein 8 (ORP8) underlying the mechanism implicated in the obesity associated-IR [291]. Mir-143 expression proved to be deregulated in the mesenteric fat tissue of mice with high-fat-diet induced obesity. The overexpression of mir-143 was associated with leptin levels and IR. Furthermore, mir-143 disrupted the expression of PPARg and Ap2, adipocyte differentiation markers [292]. The overexpression of mir-143 was also identified in obese and morbidly obese patients [289,293]. Furthermore, the expression of mir-143 proved to be deregulated in the pediatric population suffering from obesity and its low levels were associated with disturbances in the lipid metabolism [289]. On the contrary, in the study conducted by Viesti A Collares R, no difference was identified regarding mir-143 expression between obese and non-obese patients [294].

While the overexpression of mir-143 is closely associated with IR [292] and obesity [291], its implication in oncogenesis was also established firstly through the inhibitory effect over the expression of *Bcl2, extracellular signal-regulated kinase-5(ERK5), and KRAS* [178]. Mir-143 proved to have antitumor effects in BC cells, thus abolishing the cancer cells growth by reducing the expression of *ERBB3* [179], *Kras, Vimentin, CXCR4, MMP-9* and increasing the expression of E-*Cadherin* [180]. Moreover, it acts synergically with miR-145, thus the cluster miR-143/145 exerts a greater anti-tumor effect than each individual miR [179].

The expression of miR-143 was also downregulated in ESCC. Mir-143 exerts its tumor suppressor capacities by targeting FAM83F and by inhibiting its activity, thus possessing anti-proliferative affects in ESCC cells [181]. Furthermore, miR-143 proved an anti-cancerous effect in gastric cancer (via DNMT3A) [182]. It has been shown that miR-143 has different expression patterns in colon vs. rectal cancer although the clinical implication of this phenomenon is unknown [295]. In the case of hepatocellular carcinoma, miR-143 regulates a plethora of genes. Thus, miR-143 downregulates the expression of *TLR2, NF-κB, MMP-2, MMP-9, CD44, MMP14, integrin β1,* and *integrin β4* and upregulates the expression of E-*cadherin* [183].

Regarding osteosarcoma, miR-143 reduces the tumors’ capacity for lung metastasis with no influence on the neoplastic cell proliferation rate, mainly through the downregulation of *PAI*-1 [184].

TGF-B upregulates the miR-143 expression in NSCLC. Additionally, miR-143 suppressed the migration and invasion of NSCLC cells [185].

Taking into consideration the aforementioned findings, it is obvious that KRAS represents a crucial target that links the tumor suppressor miR-143 and various types of cancers (cervical cancer, prostate cancer, CRC, breast cancer NSCLC). KRAS is implicated in a plethora of essential pathways implicated in cancer cells proliferation, angiogenesis, invasion, and dissemination [296].

**MiR-145**

MiR-145 regulates AKT enzyme expression, which couples PI3K and GLUT4 activation, an important part in the insulin signaling pathway. A study demonstrated that obese mice present upregulation of miR-145, which prevents insulin-stimulated AKT activation [41]. Kirby et al. subsequently demonstrated in a study evaluating the expression of some miRs in subcutaneous adipose tissue in individuals with preserved insulin sensitivity and in persons with insulin resistance that miR-145 expression is at least three times lower in insulin resistant persons. In the same study, the authors evidence the target genes of miR-145: *ADAM22, MYO5A, LOX,* and *GM2A* [297].

Studies have shown that miR-145, along with miR-143, plays a role in controlling vascular homeostasis by regulating smooth muscle cell plasticity and responsiveness to the action of ACE (angiotensin-converting enzyme), which suggests a possible connection between the imbalance of miR-145/143 associated with obesity, increased cardiovascular risk, and poor blood pressure control, representing in this way a potential therapeutic target. It should be mentioned that low miR-143/145 expression was observed in several types of cancer, which might limit therapeutic potential in metabolic diseases [291].

MiR-145 is one of the most studied miRs in cancer, being involved in tumor proliferation, differentiation, apoptosis, metastasis, angiogenesis processes, as well as in therapeutic resistance. A meta-analysis showed that low miR-145 expression is associated with shorter OS in ovarian, CRC, glioma, osteosarcoma [186].

Like in the case of other miRs, the actions of miR-145 are multiple. In urinary bladder urothelial carcinoma, it has a suppressive action by acting on *KLF4*, which disturbs the Warburg effect and induces cell proliferation inhibition. Suppressive action has also been demonstrated in ESCC through the action on c-*Myc*, and in gastric cancer where miR-145 suppression increases *Ets1* expression, facilitating in this way tumor metastasis and angiogenesis. In TNBC, it plays a role in cell adhesion, regulating the activity of E-cadherin. Certain targets of miR-145 are involved in the pathogenesis of some cancers. Through the effect on the *FSCN1* gene, it is involved in the tumorigenesis of urinary bladder, esophageal, nasopharyngeal, liver, prostate cancer [186].

It has been demonstrated that miR-145 overexpression can increase the efficacy of chemotherapy. For example, in BC, miR-145 may induce intracellular doxorubicin accumulation through the suppressive effect on MRP1. The inhibition of the PI3K/AKT signaling pathway, which in turn induces MRP1 and P-gp inhibition, increases the sensitivity of esophageal squamous cell carcinoma to cisplatin and that of CRC to oxaliplatin. An increase in tumor cell sensitivity to the cytotoxic action of cetuximab in CRC was also observed, through a reduction of BCL2 and an increase in the activity of caspases 3/7 [48].

Noteworthy is the implication of mir-145 in the development of breast cancer associated with T2D [298]. The mir-145/PI3K/Akt axis might represent a common pathway that links mir-145 expression to both MetS and cancer, taking into consideration its implication in the insulin signaling pathway [41] and in cancer pathogenesis [48].

**MIR-155**

MiR-155 is implicated in the glucose metabolism via C/EBPβand HDAC4. Overexpression of miR-155 was associated with an improvement of glucose serum levels. Additionally, the miR-155 knockout not only causes hyperglycemia but also increases the IR [299]. Upregulated miR-155 levels were also identified in the IR associated with Polycystic Ovary Syndrome (PCOS) model in rats. The IL-6/pSTAT3/miR-155/miR-21/PPAR-c pathway might represent the core mechanism which underlies the PCOS-associated IR [90].

MiR-155 has been found to be implicated in diet-induced obesity. Thus, miR-155 loss in mice resulted in less weight gain associated with a high-fat diet due to the upregulation of several genes implicated in adipogenesis (*Creb1, Cebpb, Pnpla2, Fabp4, Fasn, Ucp1, Cidea, PPARg*), insulin sensitivity (*Irs1, Glut4*), and inflammation (*AdipoQ*) [300]. A higher expression of miR-155, induced by NF-kB/TNF-alpha, was detected in subjects with obesity. Concerning the chronic inflammatory state associated with obesity, miR-155 mediates this process by inducing the expression of a plethora of genes and chemokines [301]. The adipose tissue associated macrophages represent a cellular source of miR-155 in the fat tissue [302]. In obese mice, ATM expressed higher levels of intracellular mir-155. The higher expression of miR-155 led to deregulations in the glucose metabolism, decreasing the glucose cellular uptake [7]. An interesting finding is that miR-155 may play a role in the mechanisms underlaying the obesity paradox [303].

The increased expression of oncomiR-155 in the osteosarcoma cancer cells was associated with the upregulation of several cancer stem cell surface markers, transcriptional factors, and Actinomycin D treatment resistance. Additionally, a positive feedback loop was identified between TNF-alpha and miR-155 which resulted in increased cancer cells aggressiveness [189]. MiR-155 downregulation suppresses cell proliferation and leads to cell programmed death via the NF-kB pathway [190].

OncomiR-155 plays a part in the breast cancers’ pathophysiological process. Thus, miR-155 was correlated with tumor-associated inflammation, metastatic adenopathy [191], and aggressiveness [192].

The overexpression of oncomiR-155 in gallbladder cancer was demonstrated and proved to be a marker of tumors’ aggressiveness and unfavorable prognosis being associated with cancers’ progression and lymph-node metastasis [193].

The implication of miR-155 was also observed in other types of cancers, for example: uveal melanoma via Nedd4-family interacting protein 1 [194], esophageal cancer via FGF2 [195], nasopharyngeal carcinoma [196], and colon cancer [197].

A common target of mir-155 that links MetS/obesity and cancer is *PPARg*. *PPARg* mediates the mir-155 effect on adipose cells. Thus, mir-155, by targeting the PPAR mRNA 3′UTR, regulates the chemokine expression in adipocytes, the overexpression of mir-155 being associated with the adipose tissue inflammation [282]. Furthermore, the downregulation of *PPARg* disrupts the metabolic homeostasis in adipose tissue and favours the beige/brown differentiation of fat cells. More important is that the upregulation of *PPARg* proved to be helpful in combating breast cancer-associated cachexia [304].

**MiR-193b**

High values of miR-193b were detected in the serum of persons with IR compared to the serum of subjects with preserved insulin sensitivity, as well as in patients with prediabetes compared to those with T2D, suggesting the utility of this biomarker in the early diagnosis of these disorders [305,306]. MiR-193b is expressed in high amounts in adipose tissue, where it contributes to the differentiation of brown adipocytes and to the decrease of inflammation through its inhibitory action on CCL-2 (chemokine C-C motif ligand 2), a key factor involved in inflammation associated with obesity. Low MiR-193b expression was found in subcutaneous adipose tissue of people with obesity [177].

Regarding its implication in carcinogenesis, miR-193b seems to play a dual role, acting as an oncogene in some types of cancers and as a tumor suppressor in others [207].

Several molecular pathways have been proposed to explain the pathogenic role of miR-193 in cancer. For example, in triple negative breast carcinoma, there is a low expression of miR-193b, which is correlated with a high expression of its target DDAH1, a protein with an important role in tumor angiogenesis [206]. In CRC and gastric adenocarcinoma, miR-193 acts on the TGF-beta signaling pathway, with a role in cell proliferation and apoptosis. In SCCHN, miR-193b acts as an oncogene through its action on NF1, and in pancreatic cancer, it acts as a tumor suppressor by directly targeting KRAS through AKT, ERK, and MAPK pathways [207].

**MiR-181c-3p**

MiR-181c-3p is a member of the miR-181 family and has been considered to be a novel tumor-associated miRNA in recent years. The role of miR-181c in cancer progression is controversial [77].

MiR-181c commonly appears to be a suppressing factor in various malignancies. In gastric cancer, miR-181c is significantly down-regulated and correlates with a relatively poor prognosis [201]. In breast cancer, miR-181c is the up-streaming regulator of PPAR-α implicated in EMT, being remarkably decreased in cancer cells [77]. Down-regulation of PPARα was significant, while expression of miR-181c-3p was induced by ectopically using miR-181c-3p mimic. Based on our study, miR-181c-3p would be considered as a tumor suppressor miRNA, and *PPARα* as a direct target gene for miR-181c-3p.

According to Wang, *SPP1* is one of the genes likely to participate in the enhancement of HCC growth, which provides a new potential target for the prevention and treatment of HCC. Furthermore, miR-181c in HCC cells presents characteristic direct interaction with *SPP1* as an up-streaming inhibitor, which strongly suggests new strategies in HCC research and treatment for establishing interventional practice at the molecular level.

Zhang et al. demonstrated the role of miR-181 oncomir by suppression of *PTEN* in breast cancer [198]. In pancreatic cancer, miR-181c is significantly increased [198].

Liu et al. demonstrated that overexpression of miR-181 in A549/DDP cells induced apoptosis and autophagy, reducing cell proliferation and migration via the PTEN/PI3K/AKT/mTOR pathway [307]. Thus, miR-181 could be useful in elucidating the potential molecular mechanisms underlying chemotherapy drug resistance in NSCLC, providing a foundation for novel therapeutic strategies for the treatment of NSCLC in the clinical setting.

**MiR-210**

In patients suffering from obesity, mir-210 is overexpressed in ATM [283]. Moreover, miR-210 resulted from ATM is implicated in the pathogenesis of diabetes in obese mouse models by altering glucose uptake and mitochondrial complex IV activity by targeting NADH dehydrogenase ubiquinone 1 alpha subcomplex 4 gene [308].

The mir-210 disrupted mitochondrial metabolic functions might also be implicated in cancer. GPD1L is a direct target of mir-210, which is implicated in mitochondrial homeostasis in hypoxic conditions. A valid hypothesis is that mir-210 might adjust its functions according to the oxygenation of the tumor microenvironment. Thus, it might act as a tumor suppressor in the initial stages of tumor growth and as an oncomir as the cancerous process evolves and the hypoxia becomes more important [309].

MiR-210 exerts its effects in lung adenocarcinoma cells by targeting Lysyl oxidase-like 4 (LOXL4). Consequently, the tumor cells exhibit enhanced capacities of proliferation, migration, and invasion [208].

Oncomir-210 is a marker for poor prognosis in gliomas [250] and its overexpression was demonstrated in osteosarcoma cells [210].

In pancreatic cancer cells, an increased expression of mir-210 under hypoxic conditions can be noticed. Additionally, hypoxia conditions cause gemcitabine treatment resistance and epithelial–mesenchymal transition [211].

**MiR-221**

Decreased plasmatic levels of miR-221 in obese subjects have been described in several studies [310,311]. Furthermore, reduced levels of miR-221 were associated with gestational obesity [312]. The pattern of miR-221 expression among morbidly obese patients changed once the weight loss surgery was performed. Thus, the levels of miR-221 were upregulated in patients that underwent gastric-bypass surgery [310]. The dysregulation of circulating miRs in obesity and diabetes has been further assessed by Nunez Lopez et al. [313].

By reducing Sirtuin-1 (SIRT1) protein levels, miR-221 induces white adipose tissue inflammation and IR [314]. Palmitic acid upregulated the expression of miR-221 which consequently disturbed the IRS/PI3K/AKT signaling in the initial phases of IR [42].

MiR-221 could be responsible for tumor necrosis factor-related apoptosis-inducing ligand (TRAIL) resistance in breast cancer cells by regulating *PTEN*, thus inducing EMT and increasing migration and invasiveness of breast cancer cells. Interestingly, TRAIL sensitization and a reduction of migration abilities and invasiveness of the neoplastic cells were noticed after oncomir-221 knockdown [212]. MiR-221 is implicated in trastuzumab resistance of the HER2 -positive breast cancer cell line by targeting *PTEN*. On the other hand, the *PTEN* overexpression reversed the trastuzumab resistance in breast cancer cells and suppressed their invasion capacities [213].

The link between estrogen-receptor-alpha (ERα), progesterone receptor (PR), hypoxia-inducible factor 1-alpha (HIF1-α), SLUG, and miR-221 circuit was also investigated in obese and nonobese women diagnosed with endometrial cancer [214], but further studies are needed in this regard.

OSCC invasion and migration were augmented by the direct interaction of miR-221 and its target 3-UTR of methyl-CpG binding domain protein 2 (MBD2) with a consequent reduction of the MBD2 protein [215].

The profile of miRs regulating the TNF-related apoptosis-inducing ligand (TRAIL) in NSCL revealed that TRAIL resistant cells overexpressed five miRs among which are miR-221 and -222. TRAIL-induced apoptotic cell death proved to be mediated by the oncomirs-221 and -222 and their target 3-UTR of Kit and p27 (kip1) mRNAs [216].

The overexpression of miR-221 in osteosarcoma cells increased their aggressiveness not only by enhancing their proliferation and migration abilities but also by augmenting their invasiveness. The underlying mechanism that may explain this process is PTEN suppression [217]. MiR-221 is responsible for cisplatin resistance in osteosarcoma cells through protein phosphatase 2 regulatory subunit B alpha (PPP2R2A) downregulation [218].

The tumor microenvironment plays a decisive role in cancer progression and metastasis as shown in the study of the interactions between extracellular vesicles (EVs)-derived oncomirs, HCC cells, and cancer-associated hepatic stellate cells (caHSCs). In this regard, miR-221 together with miR-21 and -151 proved to have an oncogenic effect on HHC cells by modulating the protein kinase B (AKT)/extracellular signal-regulated kinase (ERK) pathways [100].

The oncomiR-221 upregulation is associated with the gastric cancer’ progression, invasiveness, lymph node metastasis, and an overall poor prognostic [315].

Regarding prostate cancer, there is a cluster of miRs (miR-20a, miR-21, miR-145, and miR-221) which proved to be helpful in the differentiation between high risk and low risk patients concerning the aggressiveness of prostate cancer [89]. Interestingly, the miR-221 expression in prostate cancer patients is reduced by the AR agonists (mibolerone (MIB) and dihydrotestosterone (DHT)) [316]. This unpredictable effect should be evaluated in future studies in order to establish the clinical implications of these findings.

SRT1 represents the common pathway that links cancer and IR/adipose tissue inflammation. Thus, an increased expression of mir-221 not only decreases the SIRT1 protein level resulting in IR and inflammation in adipocytes [314] but also regulates the prostate cancer progression. Even though SIRT1 does not represent a direct target of mir-221, in prostate cancer cell transfected with mir-221, the inhibitor SIRT1 protein was up-regulated [317].

**MiR-222**

MiR-222 is not only overexpressed in adult patients with obesity [310], but also in obese children and adolescents [289,318]. Moreover, the alterations in the expression of miR-222 in those patients are associated with imbalances of MetS biological markers [286]. T2D was also associated with disturbances in the circulating levels of miR-222. Noteworthy is the fact that insulin infusion reduced the circulating levels of miR-222. Furthermore, miR-222 levels were inversely correlated with the metformin dose administrated in T2D subjects [319]. De Mendonça M. et al. found that miR-222 mediates the effect of pioglitazone on insulin sensitivity in skeletal muscle of diet-induced obese mice, independent of PPAR [320].

In placenta and/or pancreatic tissues of patients and animal models with gestational diabetes (GDM), the expression of miR-222 and NLR family pyrin domain containing 3 (NLRP3) inflammasomes were up-regulated while C-X-C chemokine receptor type 4 (CXCR4) was downregulated. Improvement of insulin sensitivity in GDM mice through the inhibition of miR-222 together with the overexpression of CXCR4 was noticed. The upregulation of miR-222 together with the downregulation of glucose transporter 4 (GLUT4) and estrogen receptors (ERs) were strongly correlated with the serum concentration of estradiol. Thus, the hypothesis that the action of miR-222 on GLUT4 and ERs is responsible for the estrogen-induced IR [321].

MiR-222 promotes the proliferation of preadipocytes and the accumulation of lipids in mature adipocytes by inhibiting the lipolysis. Increasing evidence links miR-222 to MetS, making it a valuable potential therapeutic target in the management of obesity and IR [322].

The mir-222/CXCR4 pathway is not only implicated in GDM but also in breast cancer. Mir-222 expression was downregulated in breast cancer-associated TAMs. Mir-222 regulates the macrophage migration in breast cancer through the CXCR4 pathway. Mir-222 is inversely correlated with TAM chemotaxis [323]. OncomiR-222 overexpression proved to increase colon cancer cell aggressiveness by promoting their migration and invasion abilities. MiR-222 alters the colon cancer cell migration through the downregulation of its target gene mammalian STE20-like protein kinase 3 (MST3) which plays a key role in the phosphorylation of paxillin, thus reducing the intercellular adhesion. Furthermore, miR-222 together with MST3 play a crucial role in the production of inadopodia [219]. Furthermore, the overexpression of miR-222 in aggressive papillary thyroid cancer tissues was established. In vitro studies revealed that miR-222 exerts its effects via 3′-UTR of protein phosphatase 2 regulatory subunit B alpha (PPP2R2A)*,* thus enhancing the invasiveness and the migration of thyroid cancer cells. The AKT signaling pathway also proved to play a role in miR-222-mediated invasion and metastasis of papillary thyroid cancer [220]. As in colon cancer, miR-222 proved to enhance the formation of lung metastasis in thyroid cancer patients [219,220].

**Mir-221/222**

The miR-221/222 cluster is implicated in both IR and breast cancer pathogenesis through its downregulating effect on CAV1. Mir-221/222-induced deregulation of CAV1 represents a key pathway involved in breast cancers’ invasion, migration, and metastasis [51].

Furthermore, the cluster mir-222/221 is implicated in both IR and cancer by targeting genes that are implicated in both MetS and cancer pathogenesis: *transcription factor v-ets erythroblastosis virus E26 oncogene homolog 1 (ETS1), DICER, PTEN* [324].

Regarding the implication of miR-221/-222 in the cancers’ pathological process, it targets the following genes *p27Kip1, CDKN1C/p57, E-cadherin, PTPµ, PUMA, ARID1A, AHR1* [324].

Cancer lack of responsiveness to treatment remains an ongoing impediment for clinicians. The aggressiveness of this disease is in part the result of miR-221/-222-mediated signaling by targeting β4 integrin, STAT5A, and ADAM-17 [221]. Thus, the role of the miR-221/-222 cluster and its target genes (*p27, p57, estrogen receptor alpha*) in BC cells survival and the lack of response to estrogen was established [222]. The increased expression of miR-221/-222 in breast cancer cells enhances tumor aggressiveness through the activation of Wnt/β-catenin signaling by downregulating the target genes *WIF1, DKK2, SFRP2,* and *AXIN2* [223].

Deregulation of miR-221/-222 is also implicated in other solid malignancies such as gliomas, by targeting TIMP2 [224] and retinoblastomas [225].

**MiR-365**

Along with miR-193b, miR-365 plays an important role in differentiation of brown adipocytes. Recently, it has been demonstrated that brown adipose tissue (BAT) plays a more important role in humans than was initially considered. The amount of BAT is inversely correlated with BMI and the basal metabolic rate. The decrease in BAT activity may contribute to the development of obesity and IR. In vitro inhibition of miR-193a/b and miR-365 expression inhibits brown adipocyte differentiation as a result of the inhibition of some key genes involved in adipogenesis such as *adiponectin*, *Cebpα, Fabp4*, and *Pparγ* [325].

In pancreatic ductal adenocarcinoma, miR-365 values were associated with the therapeutic response [230]. In NSCLC, the values of miR-365 are correlated with prognosis, and this is involved in tumor pathogenesis through its action on TTF1, ETS1, PTEN [231]. In triple-negative breast cancer, miR-365 inhibits tumor proliferation, migration, and invasion through its action on ADAM1, the miR-365/ADAM1 axis being suggested as a possible therapeutic target [232].

**MIR-375**

MiR-375 is one of the miRs specific to pancreatic beta cells with a role in the suppression of glucose-stimulated insulin secretion through inhibition of myotrophin expression. Additionally, it plays an important role in glucose homeostasis, cell turnover, and in the differentiation of pancreatic beta cells [326]. In addition, it stimulates adipogenesis in preadipocytes by regulation of the ERK1/2 signaling pathway [327].

Some studies showed a significant increase in miR-375 in the plasma of patients with T2D compared to normoglycemic persons, suggesting its potential utility as a biomarker [272].

Numerous studies demonstrated the implication of miR-345 in the process of carcinogenesis. For example, miR-375 inhibits AEG-1 oncogene expression, and low miR-375 values accompanied by AEG-1 overexpression are involved in tumor growth and invasion in HCC and head and neck cancers [234,235]. In gastric cancer, low expression stimulates cell proliferation by attenuating the effect on the JAK2 signaling pathway and through the action on PDK1 and YWHAZ [236]. The tumor suppressive effect of miR-365 in esophageal squamous cell carcinoma was demonstrated in vivo and in vitro by identifying IGF1R as a target of miR-365, an important component of the PI3K-AKT/PKB pathway. Other cancers in which a low miR-365 expression was observed are uterine cervical cancer through the action on SP1, prostate cancer, CRC, melanoma, pancreatic cancer [233].

**Let-7**

Let-7 is downregulated in obesity and it targets HMGA2 [328]. The let-7 family is involved in adipocyte differentiation by targeting the high-mobility group AT-Hook 2 (HMGA2) protein, which reduces adipose tissue in obese leptin-deficient mice [329], suggesting, once again, a role for leptin and obesity in CRC [330].

In breast cancer, many studies have shown that let-7 inhibits HMGA2, MYC, JAK-STAT-3, caspase-3, RAS, CCND2, Erα [331,332,333].

In lung cancer, let-7 miRNA expression levels are changed [237] and low let-7 expression is significantly associated with shorter postoperative survival. In contrast, the study of Inamura [238] shows that decreased expression of let-7 occurs early during tumor progression and does not correlate with prognosis of bronchioloalveolar carcinoma. In lung cancers, up-regulation of HMGA2 and down-regulation of let-7 has been reported [240]. The effect of let-7 on HMGA2 was determined by multiple target sites in the 3’ untranslated region (UTR), and overexpression of the HMGA2 ORF without a 3’UTR rescued the growth-suppressive effect of let-7 on lung cancer cells. These results offer a novel example of suppression of an oncogene by a tumor-suppressive miRNA and indicate that the oncogene is activated by some tumors through chromosomal translocations that remove the oncogene’s 3’UTR containing the let-7 target sites.

Overexpression of let-7 has been shown to inhibit proliferation of ovarian cancer [246], prostate cancer [245], colon cancer [242], osteosarcoma [249], and neuroblastoma [247]. Several important cell cycle regulators including cyclins, cyclin-dependent kinases (CDKs), Ras, HMGA2, *MYCN*, and c-Myc have been confirmed to be targets of let-7 (see Table 1).

Let-7 functions as an onco-miR in CRC [241].

The important roles played by LIN28/let-7 in tumor progression involve this pathway as an attractive therapeutic target. Reversal of LIN28 expression in a full-blown tumor has been demonstrated to induce tumor cell differentiation and decreased tumor invasiveness, and antagonizing LIN28 would induce tumor cell differentiation and might have beneficial effects alongside chemotherapy, given that well-differentiated tumors are generally less aggressive and less drug-resistant, having better clinical outcomes [245]. He et al. [243] showed that the *PVT1-214*/Lin28/let-7 axis performs the function of a critical regulator of CRC pathogenesis, which may provide a new direction for the development of CRC therapy.

## 3. Conclusions

Obesity/Mets can induce cancer by deregulation of several miRs that are involved in metabolic processes, inflammation, and proliferation signaling. On the other side, different miRs are deregulated in cancer patients with comorbid obesity/MS, suggesting that there are some sharing mechanisms involved in adipogenesis and carcinogenesis. Currently, there is no single miR that can predict the prognosis or serve as a single biomarker. Some combinations of miRs have the potential to become prognostic markers, specific to different types of cancer, but this possibility needs to be further explored and validated.

Based on the tight connection between cancer and inflammation, targeting the inflammatory factors of the tumor microenvironment is a promising strategy for cancer prevention and treatment.

Modulation of these miRs with mimics or inhibitors could serve as a promising cancer gene therapy for tumor control and metastasis inhibition.

A variety of dietary compounds and supplements found in cruciferous vegetables, green tea, soya, turmeric, red grapes, blueberries, and spices like curry and black pepper proved beneficial in cancer prevention by modulation of microRNAs [334]. They are able to modify the epigenome and can be incorporated into the ‘epigenetic diet’ to protect against cancer and the aging process.

## Figures and Tables

**Table 1:** Adiposity-related miRs with potential role in cancer.

miRNAs	Target Genes and Functions in Obesity/IR/MeS	Target Genes or Pathway in Cancer	Type of Cancer (Oncomir/Tumor Suppressor)	References for Cancer Genes or Pathway
miR-21	TIMP3 PPAR-c, GLUT4 PTEN/PI3K/Akt pathway; STAT3, TGFRB2, PTEN, Sprouty1 and Sprouty 2	RhoB	HCC, Breast cancer ↑	[96]
		PDCD4 and Maspin	Prostate cancer ↑	[97,98]
			Prostate cancer ↓	[99]
		AKT/ERK pathways	HCC ↑	[100]
		RECK	Osteosarcoma ↑	[101]
		CASC7 and IGN3	Colorectal cancer ↑	[102]
		PTEN	Colorectal cancer ↑	[103]
		RhoB	Colorectal cancer ↑	[104]
		Sec23A	Colorectal cancer ↑	[105]
		TIMP-3 and RECK	Colorectal cancer ↑	[105]
		STAT3, PIK3R1	Breast cancer ↑	[106,107]
		PTEN	NSCLC ↑	[108]
		VHL/PI3K/AKT	Papillary thyroid cancer ↑	[109]
		PTEN and PDCD4	Gastric cancer ↑	[110]
		TIMP3	Melanoma ↑	[111]
		Sox2/β-catenin, RECK	Glioma ↑	[112,113]
		PTEN, DKK, BCL2	OSCC ↑	[114,115,116]
		PTEN	Cervical cancer ↑	[117]
			RCC ↑	[118]
miR-24-3p	SR-B1, HMGCR, DHCR24, SREBP2 KCNQ1	LPAATβ	Osteosarcoma ↓	[119]
		CCK8	Colorectal cancer ↓	[120,121]
		p27Kip1, Bim	Breast cancer ↓	[122,123]
miR-26a	GSK3β, PKCδ, PKCθ, ACSL3, ACSL4, PCK1, TCF7L2, FXl19	p53, SMAD1, EZH2, IL-6-Stat3, CTDSP1/2/L, SODD, CKS2, FGF9	Lung, breast, HCC, rabdomyosarcoma, prostate, melanoma, papillary thyroid, gastric, pancreatic cancer ↓	[124]
		MCL-1 BRCA1	Breast cancer ↓ Triple-negative BC↓	[125] [126]
		PTEN and PHB, ERα, GSK3	Glioma, ovarian cancer, colon cancer, cholangiocarcinoma ↑	[124]
miR-26b	Glut 4, PTEN/PI3K/AK, Fbxl19	Cox-2	Glioma, NSCLC ↓	[127,128]
		EphA2	HCC ↓	[129]
miR-27	PPAR-γ, Wnt1, GLUT-4 PI3K, PRDM16, PPARα, CREB, PGC1β	ZEB1, ZEB2, Slug, Vimentin, E-cadherin	Gastric cancer ↓	[130]
		MDR1/P-glycoprotein	Cancer cells ↑	[130]
		SPRY1, BAK, FOXO1, CBLB/GRB2	Breast cancer ↓	[131,132]
miR-27b	PHB, INSR, PPARγ	PPARγ	CRC ↑	[37]
		CDH11, EMT, PPARγ- NHE1 pathway	Cervical cancer ↑	[39,133]
		LIMK1, Sp1	NSCLC ↓	[48,134]
miR-30	DDL40-Notch-1		Breast cancer ↑	[135]
		MMP19	NSCLC ↑	[136]
		Fibronectin, Vimentin, N-cadherin	Pancreatic cancer ↑	[137]
miR-31	PPARg, PRKAA1, ACACA, GLUT4, IRS1, HIF-1a	ARIDIA	HNSCC ↑	[138]
			Rectal cancer ↑	[139]
		FIH-1	CRC ↑	[140]
		RhoA, GNA13	Breast cancer ↑	[140,141]
		PPP6C	Mezothelioma ↓	[142]
		Dock1, NF-jB/Snail	Glioma ↓	[143]
		ZH2, p53 pathway	Gastric, ovarian, osteosarcoma, prostate cancer ↓	[63,144]
miR-34a	Inhibit macrophage M2 induced adipose inflammation	BCL-2 and SIRT1	Breast cancer↓	[145]
		Notch1	Breast cancer↓	[146]
		Wnt/β-catenin signaling pathway	Breast cancer↓	[147]
		Fra-1	Breast cancer↓	[148]
		MYC, P53	Breast cancer↓	[149,150]
		E2F3	Neuroblastoma ↓	[151]
		c-Met and β-catenin	Colon cancer ↓	[152]
		P53	Osteosarcoma ↓	[153]
		MET, P53	Ovarian cancer ↓	[154,155]
		CD44	Prostatic cancer ↓	[156]
		AXL	Solid cancer ↓	[157,158]
miR96	INSR, IRS	SOX6, EphrinA5	HCC ↑	[159,160]
		PTPN9, FOXO1, FOXO3a	Breast cancer ↑	[161,162,163]
		FOXO3	NSCLC ↑	[164]
		RECK	ESCC ↑	[165]
		AEG-1	Glioblastoma ↓	[166]
		Ezrin	RCC ↓	[167]
		KRAS	Pancreatic cancer ↓	[168]
miR-100	mTOR, IGFR, VLDLR		Bladder cancer ↓	[169]
		HOXA1, Rac1, ICMT, EphB6, AGO2, Plk1, Wnt, β-catenin or RBSP3	HCC, RCC, bladder cancer, NSCLC, and epithelial ovarian cancer ↓	[170] [171]
		mTOR kinase	Endometrioid endometrial carcinoma ↓	[171]
			CRC ↓	[172]
miR-125b	PI3K/AKT JNK signaling pathway, SIRTs	NF-κB,	DLBCL ↑	[173]
		MAPK11, IRF4, TET2-VEGFA	Acute leukemia ↑	[174]
		Wnt, PI3K/Akt, STAT-3, MAPK, NF-κB, p53	HCC, CRC, RCC, thyroid larynx, osteosarcoma, prostate melanoma, Ewing sarcoma, glioblastoma, gallbladder, ovarian cancer ↓	[175]
miR-126	IRS-1, CCL2	PI3K, KRAS, EGFL7, CRK, ADAM9, HOXA9, IRS-1, SOX-2, SLC7A5 and VEGF	Gastrointestinal tract, genital tracts, breast, thyroid, lung cancers ↓	[176]
			NSCLC ↓	[177]
			CRC ↓	[176]
			RCC ↓	[176]
miR-143	ORP8/insulin-AKT pathway, PPARg and aP2 Leptin	Bcl2, ERK5, KRAS	Cervical, prostate, CRC ↓	[178]
		ERBB3	Breast cancer ↓	[179]
		KRAS, Vimentin, CXCR4, MMP-9	Breast cancer ↓	[180]
		FAM83F	Esophageal squamous cell carcinoma ↓	[181]
		DNMT3A	Gastric cancer ↓	[182]
		TLR2, NF-κB, MMP-2, MMP-9, CD44, MMP14, integrin β1, integrin β4	HCC ↓	[183]
		PAI-1/MMP-13	Osteosarcoma ↓	[184]
		Smad3, CD44, and K-Ras	NSCLC ↓	[185]
miR-145	AKT/PI3K/GLUT4 ADAM22, MYO5A, LOX, and GM2A	PI3K/AKT, MRP1, SMAD, KLF4, c-myc, Ets1, E-cadherin, FSCN1, BCL2	BC, gastric, CRC, NSCLC, glioma, HCC, osteosarcoma, ovarian, cervical, prostate, bladder, nasopharyngeal cancer ↓	[186]
miR-148a-3p	inhibit DNMT1	DNMT1	Esophageal Cancer↓	[187]
		WNT5A, TGF-α, BTG2 and MYCBP	Chordoma ↑	[188]
miR-155	C/EBPβ, HDAC4, PPARγ, GLUT4, IRS1, PPAR-c, Creb1, Cebpb, Pparg, Pnpla2, Fabp4, Fasn, AdipoQ,	TNF∝, NF-kB pathway, ERK pathway, Caspase 3	Osteosarcoma ↑	[189] [190]
		RhoA, PEG10 and MYB	Breast cancer ↑	[191,192]
			Gallbladder cancer ↑	[193]
		NDFIP1	Melanoma ↑	[194]
		FGF2	ESCC ↑	[195]
			Nasopharyngeal carcinoma ↑	[196]
		IGF-1	Colon cancer ↑	[197]
181c-3p	PPARα; reduced inhibition of PPARα, BC proliferation	PPARα	Breast cancer ↓	[77]
		PTEN	Breast cancer ↑	[198]
		PTEN/PI3K/pAkt	CRC ↓	[199]
		PTEN/PI3K/AKT	NSCLC ↓	[200]
		XIAP, caspase 9, caspase 3	Gastric cancer ↓	[201]
		*CTGF*, *BIRC5*, *BLC2L1*	Pancreatic cancer ↑	[202]
		MGMT	Glioblastoma ↓	[203]
		SPP1	HCC ↓	[204]
		SMAD7, TGF-β	Osteosarcoma ↓	[205]
MiR-193b	CCL2, NTFYα si NRIP1	DDAH1	Triple-negative breast cancer ↓	[206]
		TGF-𝜷, SMAD3, NF1	CRC, Glioma, Head and neck SCC↑	[207]
		K-Ras, ERBB4	Lung cancer↓	[207]
		MAX, KRAS, TGF-𝜷, CCND1, ETS1, MAPK	ESCC, Gastric cancer, HCC, Pancreatic cancer ↓	[207]
		Mcl-1, c-kit, MYB	Melanoma, Leukemia ↓	[207]
		caspase 3 and 7, uPA	Ovarian, Prostate cancer ↓	[207]
miR-210	NDUFA4 GPD1L	LOXL4	Lung adenocarcinoma ↑	[208]
			Glioma ↑	[209]
			Osteosarcoma ↑	[210]
		HOXA 9	Pancreatic cancer ↑	[211]
miR-221	SIRT1 IRS/PI3K/AKT	PTEN/TRAIL	Breast cancer↑	[212,213]
		ERα, PR, HIF1-α, SLUG	Endometrial cancer↑	[214]
			Prostate cancer ↑	[98],
		MBD2	OSCC ↑	[215]
		Kit	NSCLC ↑	[216]
		PTEN, PPP2R2A	Osteosarcoma↑	[217,218]
		AKT/ERK pathway	HCC ↑	[100]
miR-222	CXCR4 GLUT4 ERs, BTG2, adipor1	p27 (kip1)	NSLCC	[216]
		MST3	CRC ↑	[219]
		PPP2R2A	Papillary thyroid cancer ↑	[220]
miR-221/222	CAV1	CAV1	Breast cancer↑	[51]
		β4 integrin, STAT5A, and ADAM-17	Breast cancer↑	[221]
		p27, p57, ER∝	Breast cancer↑	[222]
		Wnt/β-catenin, WIF1, SFRP2, DKK2, AXIN2	Breast cancer↑	[223]
		TIMP2	Gliomas ↑	[224]
			Retinoblastomas ↑	[225]
miR-302	Maintain SOX2 and c-Myc by targeting repressor of c-Myc	MACC1	HCC ↓	[83]
		Sox2, c-Myc, Nanog	Breast cancer ↑	[226]
		RUNX2	Breast cancer ↓	[227]
		TGF-β	Mucoepidermoid carcinoma of salivary glands ↑	[228]
		TGFBR2/SMAD3 RAB11A/Wnt/β-Catenin	Pituitary Tumors ↑	[229]
miR-365	Cebpα, Fabp4, and Pparγ	BTG2	Pancreas ↑	[230]
		ETS1	NSCLC ↓	[231]
		ADAM1	Triple negative breast cancer ↓	[232]
miR-375	ERK ½ Myotrophin	PSAT1	ESCC ↓	[233]
		AEG-1	HCC, Head and neck cancers ↓	[234,235]
		PDK1, YWHAZ	Gastric ↓	[236]
miR 3184-3p	FOXP4–NOTCH induced EMT	N-cadherin, vimentin, E-cadherin	Breast cancer ↓	[77]
Let 7	Inhibit HMGA2, inhibit preadipocyte proliferation, insulin-PI3K-mTOR IGF1R, INSR, IRS2	HMGA2	Breast cancer ↓	[302]
		lin-41, hbl-1/lin-57 RAS	Lung cancer ↓	[229,237,238]
		KDM3A/DCLK1/FXYD3	Lung cancer ↓	[239]
		HGMA2	Lung cancer ↓	[240]
		RAS, c-MYC	CRC ↑	[241]
		HGMA2 LIN 28	CRC ↓	[242,243,244]
		E2F2, CCND2	Prostate cancer↓	[245]
		RAS	Ovarian cancer ↓	[246]
		*MYCN*	Neuroblastoma ↓	[247,248]
		Aurora-B	Osteosarcoma ↓	[249]
